# Importance of Low- and Moderate-Grade Adverse Events in Patients' Treatment Experience and Treatment Discontinuation: An Analysis of the E1912 Trial

**DOI:** 10.1200/JCO.23.00377

**Published:** 2023-10-06

**Authors:** Nathaniel S. O'Connell, Fengmin Zhao, Ju-Whei Lee, Edward H. Ip, John Devin Peipert, Noah Graham, Mary Lou Smith, Ilana F. Gareen, Ruth C. Carlos, Samilia Obeng-Gyasi, Joseph A. Sparano, Tait D. Shanafelt, Mary L. Thomas, David Cella, Lynne I. Wagner, Robert Gray

**Affiliations:** ^1^ Wake Forest University School of Medicine, Winston-Salem, NC; ^2^ Dana Farber Cancer Institute, ECOG-ACRIN Biostatistics Center, Boston, MA; ^3^ Northwestern University Feinberg School of Medicine, Chicago, IL; ^4^ Research Advocacy Network, Chicago, IL; ^5^ Department of Epidemiology and the Center for Statistical Sciences, Brown University School of Public Health, Providence, RI; ^6^ University of Michigan Comprehensive Cancer Center, Ann Arbor, MI; ^7^ The Ohio State University, Columbus, OH; ^8^ Icahn School of Medicine at Mount Sinai, Tisch Cancer Institute, New York, NY; ^9^ Stanford Center Institute Palo Alto, Stanford, CA; ^10^ VA Palo Alto Health Care System, Palo Alto, CA

## Abstract

**PURPOSE:**

Despite defined grades of 1 to 5 for adverse events (AEs) on the basis of Common Terminology Criteria for Adverse Events criteria, mild (G1) and moderate (G2) AEs are often not reported in phase III trials. This under-reporting may inhibit our ability to understand patient toxicity burden. We analyze the relationship between the grades of AEs experienced with patient side-effect bother and treatment discontinuation**.**

**METHODS:**

We analyzed a phase III Eastern Cooperative Oncology Group-American College of Radiology Imaging Network trial with comprehensive AE data. The Likert response Functional Assessment of Cancer Therapy-GP5 item, “I am bothered by side effects of treatment” was used to define side-effect bother. Bayesian mixed models were used to assess the impact of G1 and G2 AE counts on patient side-effect bother and treatment discontinuation. AEs were further analyzed on the basis of symptomatology (symptomatic or asymptomatic). The results are given as odds ratios (ORs) and 95% credible interval (CrI).

**RESULTS:**

Each additional G1 and G2 AEs experienced during a treatment cycle increased the odds of increased self-reported patient side-effect bother by 13% (95% CrI, 1.06 to 1.21) and 35% (95% CrI, 1.19 to 1.54), respectively. Furthermore, only AEs defined as symptomatic were associated with increased side-effect bother, with asymptomatic AEs showing no association regardless of grade. Count of G2 AEs increased the odds of treatment discontinuation by 59% (95% CrI, 1.32 to 1.95), with symptomatic G2 AEs showing a stronger association (OR, 1.75; 95% CrI, 1.28 to 2.39) relative to asymptomatic G2 AEs (OR, 1.45; 95% CrI, 1.12 to 1.89).

**CONCLUSION:**

Low- and moderate-grade AEs are related to increased odds of increased patient side-effect bother and treatment discontinuation, with symptomatic AEs demonstrating greater magnitude of association than asymptomatic. Our findings suggest that limiting AE capture to grade 3+ misses important contributors to treatment side-effect bother and discontinuation.

## INTRODUCTION

In cancer trials, the Common Terminology Criteria for Adverse Events (CTCAE) guidelines established by National Cancer Institute are used to describe the toxicities patients experience and their severity. The CTCAE categorizes all adverse events (AEs) into one of 26 system organ classes in the most recent version (v5.0; eg, cardiac disorders, endocrine disorders, etc) and across five grades of severity. AE grades 1 through 5 represent mild, moderate, severe, life-threatening, and death related to AE, respectively. Documentation of AEs in clinical trials has historically relied on provider reporting. Despite five distinct grades of AEs, mild- and moderate-grade AEs (ie, grades 1 and 2) are under- or unreported, on the rationale that AEs of low and moderate grades are unlikely to affect patient safety or key trial end points.^1^ Nevertheless, while arguing that reducing monitoring for a low- or moderate-grade AE has no impact on patient safety, Mahoney et al^1^ simultaneously report that over 50% of routine AEs captured are of grade 2 or less.

**CONTEXT**

**Key Objective**
How does the co-occurrence of multiple low- and moderate-grade adverse events (AEs; ie, grade 1 and grade 2 AEs) affect patient self-reported side-effect bother and odds of treatment discontinuation because of side effects?
**Knowledge Generated**
Both the odds of increased self-reported side-effect bother and the odds of imminent treatment discontinuation because of side effects increase with the count of low- and moderate-grade AEs within a patient treatment cycle. These increased odds are greater for AEs defined as symptomatic (as opposed to asymptomatic) on the basis of criteria defined for patient self-reporting of AEs.
**Relevance *(S.B. Wheeler)***
This study illustrates the impact of mild and moderate, symptomatic AEs on patient-reported side effect burden and treatment discontinuation, underscoring the critical importance of monitoring and reporting of G1 and G2 AEs in trials.*
  *Relevance section written by *JCO* Associate Editor Stephanie B. Wheeler, PhD, MPH.

Recently, the growing interest in better understanding trial treatment tolerability and toxicity burden has resulted in more widespread patient-reported outcomes (PROs) measurement before and during treatment. Given the frequency of low-grade AEs coupled with the need to fully understand treatment tolerability and toxicity burden, routine comprehensive reporting of patients' AEs, including those considered low or moderate, is imperative. The need for comprehensive reporting of AEs is accentuated by the increasing prevalence of targeted agents and immunotherapies. In this setting where treatments are taken daily, AEs experienced may be chronic (as opposed to conventional cytotoxic agents administered once every 3-4 weeks resulting in more acutely experienced AEs) and chronic low-grade AEs may present a greater burden to patients than acute grade 3 AEs.^2,3^ The influence of AE chronicity, even of low-grade AE, on key outcomes including treatment tolerability remains to be elucidated.

Various metrics have been developed and evaluated with respect to their ability to adequately represent patient toxicity burden, including the baseline adjusted grade^4^ and the toxicity index.^5^ The latter provides a metric for calculating a single, comprehensive toxicity index score that accounts for the count of all AEs and grades experienced and has demonstrated good performance in differentiating treatment arms with respect to overall toxicity burden.^6^ From a PRO perspective, the GP5 is an emerging measure of interest. This fifth item in the Functional Assessment of Cancer Therapy (FACT-G) physical function subscale asks patients to rate their agreement with the following statement, “I am bothered by side effects of treatment” in the past 7 days using a 5-item Likert scale.^7^ The GP5 has gained attention as a possible predictor of the risk of early treatment discontinuation^8,9^ and has been shown to be significantly associated with AE grade^10^ and performance status rating.^11^

Regardless of the measurement and summary methods for toxicity burden, all methods require capturing the complete range of AE grades possibly related to treatment to fully reflect treatment tolerability. Using comprehensive AE data collected in a phase III clinical trial, we demonstrate that low- and moderate-grade AEs are important contributors to self-reported patient side-effect burden and are independently related to treatment discontinuation attributed to AEs/complications/side effects.

## METHODS

### Description of Trial Analyzed

The focus of our primary analysis is on a phase III chronic lymphocytic leukemia trial (E1912) conducted by Eastern Cooperative Oncology Group (ECOG)-American College of Radiology Imaging Network.^12^ Arm A is a chronic indefinite oral therapy (ibrutinib), and arm B is a traditional intense six monthly cycles of chemoimmunotherapy (fludarabine–cyclophosphamide–rituximab [FCR]) with treatment on once days 1-3 of every 28 days. This trial was specifically chosen for our analysis because it captured comprehensive AE data across all grades over the duration of treatment for each patient. The FACT-G questionnaire was administered several times over the duration of treatment, including once at baseline (ie, at study entry) and after cycles 3 and 6 during the induction phase of treatment. In addition, the off-treatment report form denotes the reason for treatment discontinuation, namely, discontinuation because of AEs/side effects/complications. Our analyses specifically focus on item GP5 and treatment discontinuation because of AE/side effects/complications.

### Statistical Analyses

In our primary analysis, AEs deemed unrelated or unlikely to be related to treatment were excluded. To assess the relationship between self-reported side-effect burden and AE data, in cycles in which GP5 were assessed (cycles 3 and 6), we modeled GP5 responses longitudinally in a mixed model as an ordinal response assuming a cumulative-logit link function, with the count of grade 1 and count of grade 2 AEs during the cycle included as separate continuous predictors, along with the presence of at least one grade 3 or 4 AE as a binary predictor (none *v* 1+). Regarding the latter, the count of grade 3 and 4 AEs were aggregated and included as a categorical response because of the scarcity of several grade 3+ AEs in a given patient for a given cycle. Cycle number, treatment, and baseline GP5 were included as fixed effects along with random intercepts for each patient.

To model treatment discontinuation, we used a generalized linear mixed model with a logit link function to model the log odds of treatment discontinuation, with cycle number, count of grade 1 AEs, and count of grade 2 AEs within each cycle modeled as continuous predictors and the presence of grade 3 or 4 AEs as a categorical predictor. Having previously been demonstrated as a predictor of discontinuation, baseline GP5 was included as a fixed effect,^8,9^ along with treatment, as confounders in the model, and random intercepts for each patient.

Given the outcome discontinuation being sparse, maximum likelihood–based models were unable to be reliably fit due to convergence issues. Thus, we used a Bayesian framework to model each of the generalized linear mixed models described above. All statistical analyses were performed using R version 4.10 (R Core Team; Vienna, Austria), with models fitted in STAN via the package brms for the Bayesian regression models using MCMC to obtain posterior parameter estimates.^13,14^ See the Data Supplement (Part A, online only) for further details on model specification, prior distributions, and assessment of convergence for these models. The results from the regression models are presented in terms of their estimated odds ratios estimated from the median of the posterior sample, along with the posterior 95% credible interval (CrI), with the probability of the odds ratio (OR) being different from 1 estimated from the posterior sample (denoted as Pr[OR > 1]). The latter in essence assesses the probability that a non-null association exists.

### Secondary Analysis of E1912—Assessment of Symptomatic Versus Asymptomatic AEs

In an additional analysis, we subdivided all treatment-related AEs into two classes, symptomatic and asymptomatic AEs, with symptomatic AEs on the basis of the 78 AEs identified as self-reportable for PRO-CTCAE reporting.^15^ On the basis of these criteria, we created new count variables for each AE type on the basis of whether they were symptomatic or asymptomatic (eg, count of grade 1 symptomatic AEs was a separate predictor from count of grade 1 asymptomatic AEs) and extended the models defined above to include separate variables for symptomatic and asymptomatic AEs.

### Sensitivity Analyses

We conducted multiple sensitivity analysis for our models. First, we refit our models with all AEs (regardless of treatment attribution) included in the aggregate count of AEs per patient cycle. Next, recognizing that some grade 3+ AEs may necessitate discontinuation, thus inflating the effect of the presence of grade 3+ AEs on discontinuation, we fit our treatment discontinuation model across cycles of treatment in which patients did not experience a grade 3+ AE. Thus, we assessed the effects of grade 1 and 2 AEs (at least possibly related to treatment) on treatment discontinuation because of AEs among patient cycles without grade 3+ AEs reported.

## RESULTS

A total of 529 patients were enrolled in the trial, 19 (2 in arm A and 17 in arm B) never started the protocol therapy, and at the time of this analysis, 275 had either completed or discontinued treatment, or presented with disease progression. On the basis of the study scheme, patients in arm B completed their treatment regimen per protocol after cycle 6, whereas patients in arm A were to continue maintenance therapy after cycle 7 until disease progression. Thus, the remaining 235 patients were in arm A and still in maintenance. Across the 510 patients who started protocol therapy, a total of 18,028 AEs at least possibly attributed to treatment were recorded across 8,431 patient cycles of treatment. The majority of AEs were low- and moderate-grade AEs, with grade 1 AEs making up 70.5% of all AEs (12,713), grade 2 AEs representing 19.9% (3,588), grade 3 AEs representing 8.0% (1,434), grade 4 AEs representing 1.6% (289), and grade 5 AEs representing <0.1% (4). Details on frequency of specific AE occurrence are presented in the Data Supplement (Part C).

In total, 84 patients had discontinued treatment because of AEs/side effects/complications (41.9% of 117 patients in arm A and 22.2% of 158 patients in arm B). After cycle 6, 78 of those patients reported being at least somewhat bothered by side effects of treatment. Table 1 demonstrates the mean count of each grade of AE by discontinued treatment cycles versus the majority of cycles that did not result in discontinuation because of AEs/side effects. Across all AE grades, we observed significantly more AEs in cycles preceding discontinuation because of AEs/side effects/complications reported.

**TABLE 1.**
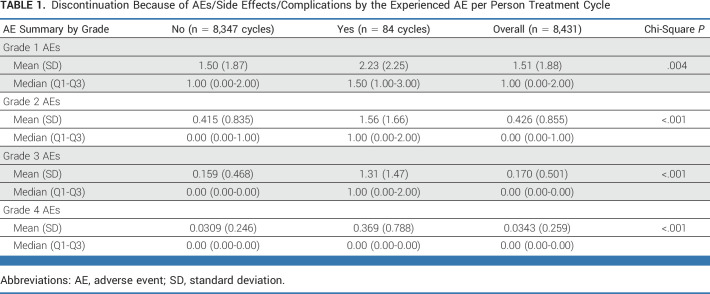
Discontinuation Because of AEs/Side Effects/Complications by the Experienced AE per Person Treatment Cycle

### Analysis of GP5 and Treatment Discontinuation by Treatment-Related AE Data

Table 2 shows results for the regression models of GP5 and treatment discontinuation on treatment-related AEs. Each model met the convergence criteria (see the Data Supplement, Part A for further details). In our cumulative logit model for GP5 response (after either cycle 3 or cycle 6), we observed that when controlling for the presence of grade 3 or 4 AEs, baseline GP5 response, treatment group, and visit, the counts of grade 1 AEs and grade 2 AEs were each independently associated with increased odds of increasing treatment side-effect bother in terms of the GP5 ordinal response. More specifically, with each additional grade 1 AE experienced, the odds of increased side-effect bother increased by 13% (95% CrI for OR, 1.06 to 1.21), and each additional grade 2 AE experienced increased the odds of increased side-effect bother by 35% (95% CrI for OR, 1.19 to 1.54), with Pr(OR > 1) >99% for both. The presence of at least one grade 3 or 4 AE increased the odds of increased side-effect bother by 39% (95% CrI, 1.02 to 1.92), with Pr(OR > 1) = 98%. Baseline GP5 was associated with follow-up GP5 responses, such that for each point increase in baseline GP5, the odds of increased follow-up GP5 increased by 87% (95% CrI, 1.21 to 2.91), with Pr(OR > 1) >99%. Treatment group B (six cycles of chemoimmunotherapy) demonstrated 56% greater odds of increased side-effect bother relative to treatment A (chronic, oral signaling inhibitor; 95% CrI, 0.86 to 2.86), with Pr(OR > 1) = 93%. Visit (ie, cycle number) demonstrated no effect on side-effect bother.

**TABLE 2.**
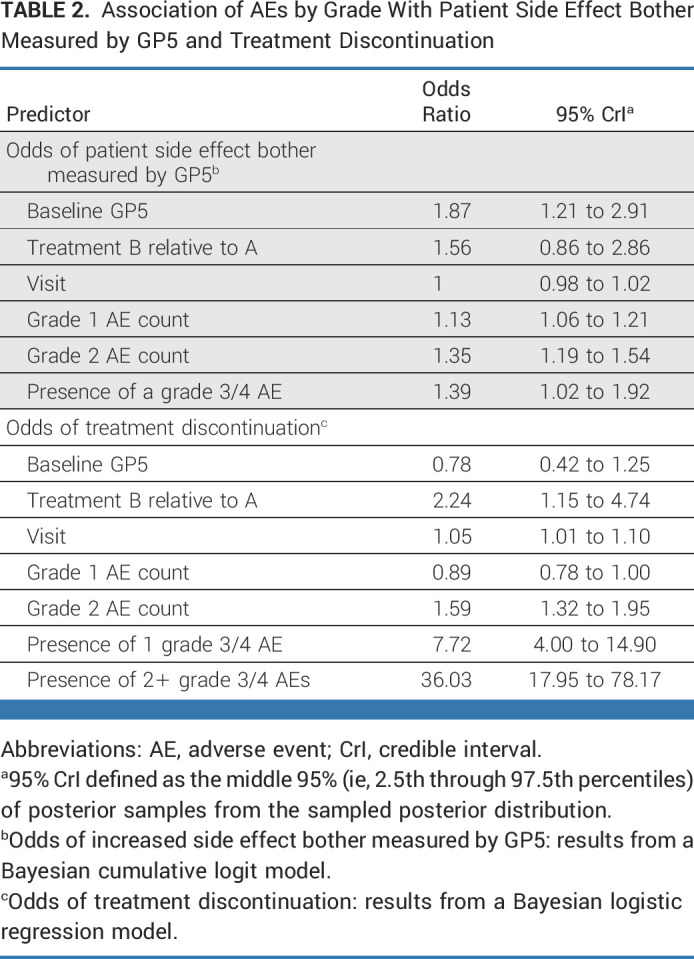
Association of AEs by Grade With Patient Side Effect Bother Measured by GP5 and Treatment Discontinuation

With respect to treatment discontinuation, adjusting for the effects of visit, baseline GP5, and treatment regimen, we observed that each additional grade 1 AE was associated with an 11% decreased odds of discontinuation (OR, 0.89; 95% CrI, 0.78 to 1.00), with Pr(OR < 1) = 97%. Conversely, each additional grade 2 AE was associated with a 59% increased odds of treatment discontinuation (95% CrI, 1.32 to 1.95), with Pr(OR > 1) >99%. Similarly, the presence of one grade 3 or 4 AE increased the odds of discontinuation by 7.72 times (95% CrI, 4.0 to 14.90), and two or more grade 3 or 4 AEs increase the odds of discontinuation because of AE/side effects substantially (OR, 36.03; 95% CrI, 17.95 to 78.17). Among the other variables we adjusted for, we found that patients had a 5% increased odds of discontinuation with each passing cycle (OR, 1.05; 95% CrI, 1.01 to 1.10), and patients on treatment B were at greater odds of discontinuation than patients on treatment A (OR, 2.24; 95% CrI, 1.15 to 4.74).

### Secondary Analyses—Differences Between Symptomatic and Asymptomatic AEs

We further examined the differential impact of treatment-related symptomatic AEs and asymptomatic AEs by grade. The results are presented in Table 3. In relation to GP5, we observed no association between the count of asymptomatic grade 1 AEs, but a 37% increased odds of increased side-effect bother for each additional grade 1 symptomatic AE (95% CrI, 1.24 to 1.52). Similarly, there was no association with asymptomatic grade 2 AEs (OR, 1.06; 95% CrI for OR, 0.89 to 1.26), but symptomatic grade 2 AEs were associated with a 106% increased odds of increased side-effect burden (95% CrI, 1.60 to 2.64) with Pr(OR > 1) >99%. The presence of a grade 3+ symptomatic AE was associated with a 5.03 times greater odds of increased side-effect bother (95% CrI, 2.15 to 11.79), with Pr(OR > 1) >99%, whereas the presence of an asymptomatic grade 3+ AE was not associated with significantly increased side-effect bother (OR, 1.21; 95% CrI, 0.86 to 1.68).

**TABLE 3.**
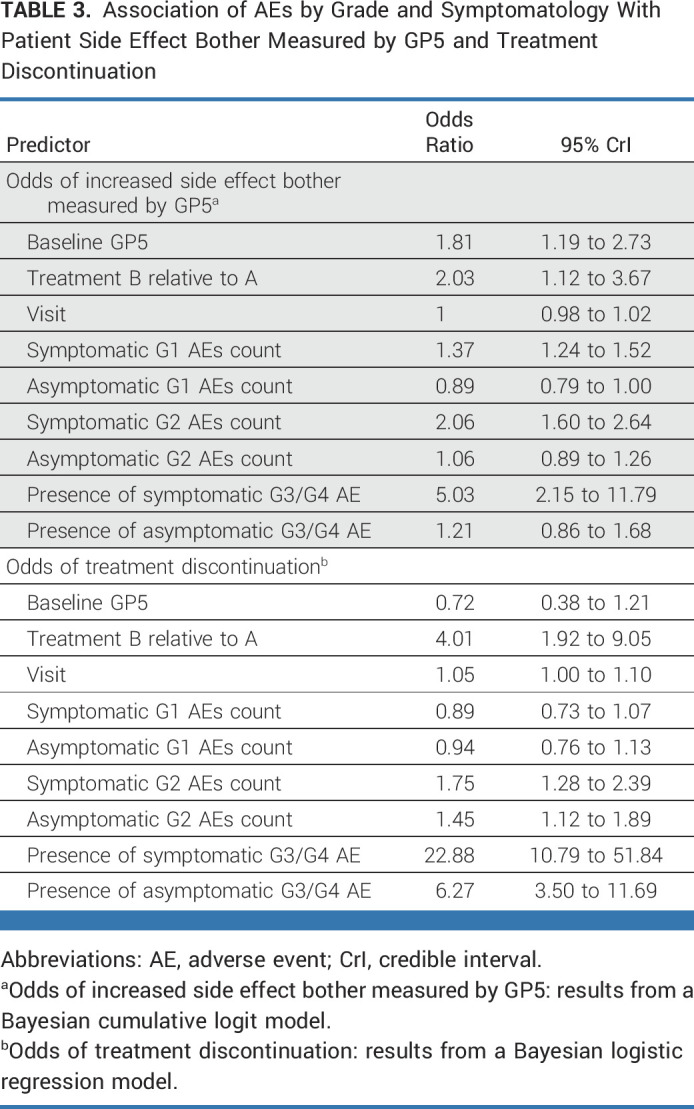
Association of AEs by Grade and Symptomatology With Patient Side Effect Bother Measured by GP5 and Treatment Discontinuation

### Sensitivity Analyses

Overall, the results from our sensitivity analyses were in line with those of our primary analyses. The inclusion of all-cause AEs resulted in similar directional associations, but of lesser magnitude with respect to the impact of grade 1 and 2 AEs on increased side-effect bother and treatment discontinuation. Among treatment cycles in which grade 3+ AEs were not present, grade 2 symptomatic and asymptomatic AEs were associated with increased odds of treatment discontinuation. More details, discussion, and tables for these analyses are presented in the Data Supplement (Part B).

## DISCUSSION

Adjusting for baseline GP5, cycle, and treatment, both the frequency of grade 1 and 2 AEs and the presence of any grade 3 or higher AEs were each independently associated with increased self-reported side-effect bother. Compared with asymptomatic AEs, an increased count of grade 2 symptomatic AEs and the presence of any grade 3 or higher symptomatic AEs were associated with increased side-effect bother. Asymptomatic AEs as defined were not associated with increased side-effect bother regardless of grade. These results suggest that symptomatic AEs as defined by the criteria of Basch et al^15^ do well in representing patient side-effect bother, whereas AEs not considered symptomatic in this way did not present a notable association with increased side-effect bother. We note that baseline side-effect bother was associated with subsequent (postbaseline) side-effect bother but not with treatment discontinuation. This finding is supported by our previous arguments that patients' level of bother reported while on trial is influenced by experiences and characteristics patients start the trial with, even before treatment, including previous experiences with cancer treatment and medications for comorbid conditions, which may lower a patient's treatment tolerability threshold.^8,16-18^

When adjusted for high-grade AEs, increased frequency of grade 2 AEs was associated with increased odds of treatment discontinuation with high probability. More specifically, both symptomatic and asymptomatic grade 2 AE frequencies were independently associated with increased odds of treatment discontinuation although symptomatic AEs demonstrated a larger estimated effect.

These results support the need for collection of all grades of AEs, especially those considered symptomatic, to facilitate understanding of patient-reported side-effect bother. Collecting patient AEs across all grades may allow us to infer patient burden in cycles when PROs are not formally ascertained, and the information gained may provide invaluable insight into patient behavioral response to treatment bother. Namely, by identifying patients during treatment with high side-effect burden including a high count of AEs of lower grades, early supportive care could be pursued to improve patient quality of life (QOL) and attempt to prevent treatment discontinuation.

Surprisingly, we observed an inverse relationship with grade 1 AEs such that an increasing count of grade 1 AEs were associated with a decreased odds of discontinuation. A possible explanation could be that the presence of AEs is associated with treatment efficacy, and thus, patients experiencing a significant count of mild AEs are less likely to discontinue because of the perception of therapeutic efficacy.^19,20^ However, the relationship shifts as the perceived benefit of treatment is reduced when experiencing multiple grade 2+ AEs.

A primary limitation of this work is the fact that our analysis was limited to a single trial data set. Unfortunately, trials with comprehensive AE data collection such as the one analyzed are uncommon. Similar analyses with more trial data sets need to be conducted to further solidify our understanding of the role of low-grade AEs in patient side-effect burden. A second limitation is that these analyses are agnostic to prolonged AEs experienced across consecutive cycles to treatment, thus ignoring that AE burden may compound over multiple cycles. Finally, we do not assess the differential impact of specific AE types on bother or discontinuation. Assessing such associations is complex and extends beyond the scope of our work, but future work could address this issue using methods such as that of Ip et al.^21^

In conclusion, these results, on the basis of a single trial, demonstrate the importance of collecting AE data across all grades, particularly symptomatic AEs. Ignoring low- and moderate-grade AEs may inhibit us from truly understanding patient burden in cancer trials and what drives them to discontinue treatment, particularly as regimens continue to shift toward targeted therapies with different toxicity profiles compared with conventional chemotherapy. Comprehensive AE data may allow us to eventually develop models to understand and predict which patients may be developing high side-effect burden and who may be at higher risk of discontinuing treatment. This in turn facilitates early supportive care to improve patient QOL and increase treatment adherence.

We acknowledge that comprehensive AE data capture across all grades may be time-consuming and impose an increased burden across study sites. There certainly exists a trade-off between this burden and the utility of the data captured, and whether this trade-off makes sense may depend on the research goals of the trial, disease, patient population, and/or treatment. Future work could further explore the costs and benefits of this trade-off and develop guidelines for all-grade AE data collection.

Future work should continue to perform similar analysis of trials with comprehensive data as we seek to better define and understand patient tolerability and its relationship with the AEs patients experience. Future work with larger trials or meta-analyses could be performed to better understand how different types of AEs, particularly those who are symptomatic, differentially affect patient side-effect burden and discontinuation.
